# Supplementary material for: Selective dependence on IL-7 for antigen-specific CD8 T cell responses during airway influenza infection
Source: Sci Rep. 2022 Jan 7;12:135. doi: 10.1038/s41598-021-03936-y (PMC8741933; doi:10.1038/s41598-021-03936-y)
Supplement: Supplementary file 1 — Supplementary Information. [file 41598_2021_3936_MOESM1_ESM.pdf]

Supplementary Figure 1

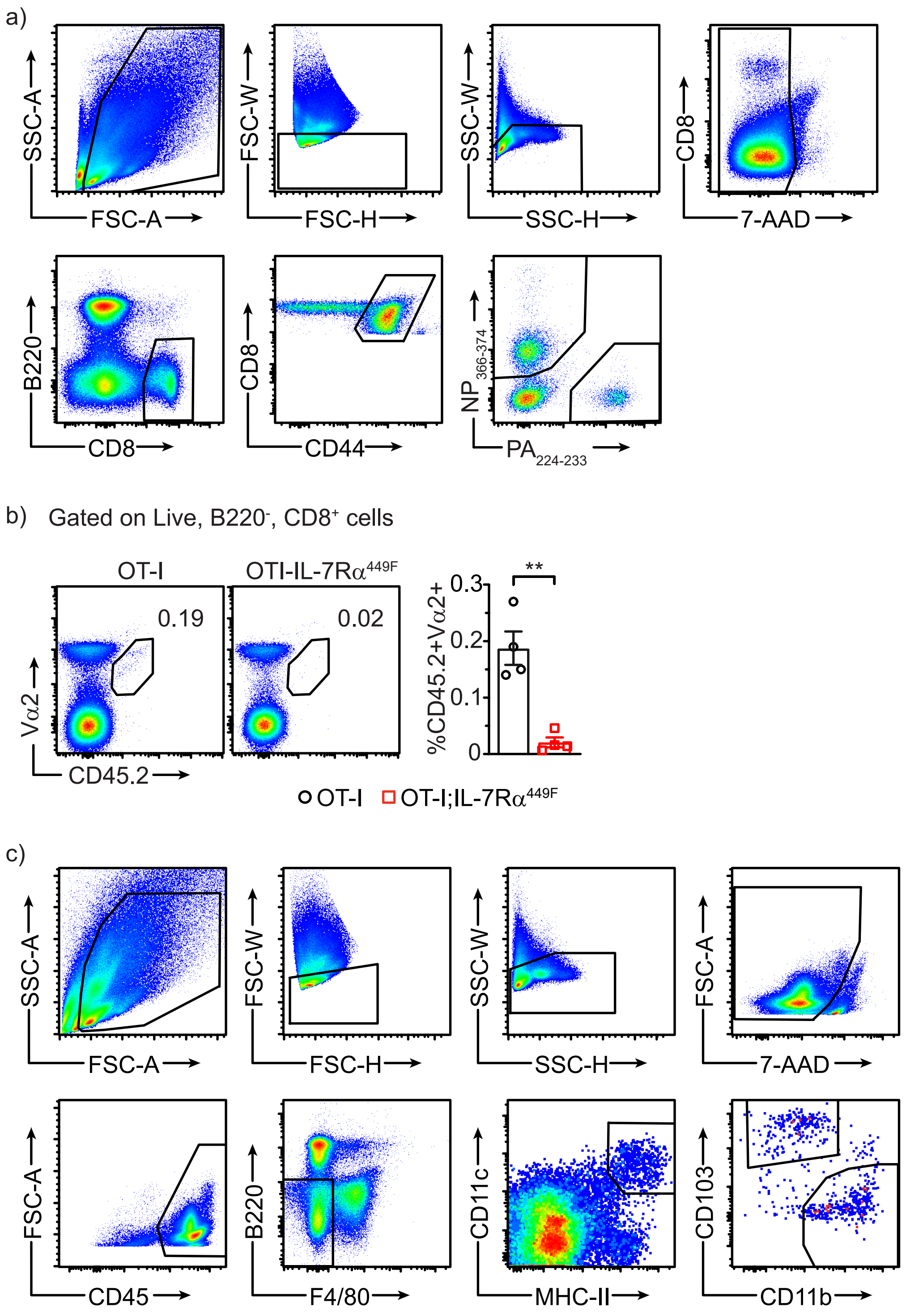

Supplementary Figure 1. (a) Gating strategy for lung and mdLN influenza-specific CD8 T cells demonstrated in lung tissues. (b) Expansion of adoptively transferred OTI-IL-7Rα^{449F} CD8 T cells is impaired in the mdLN following influenza infection as early as 3 dpi. Scatter plot and representative bar graph of CD45.2^{+} Vα2^{+} CD8 T cells. Gated within Live B220^{-} CD8^{+} cells. Datum is representative of a single experiment with n=4. **P<0.01 as determined by two-tailed Student's t-test. (c) Gating strategy for lung and mdLN dendritic cells demonstrated in lung tissues.

Supplementary Figure 2

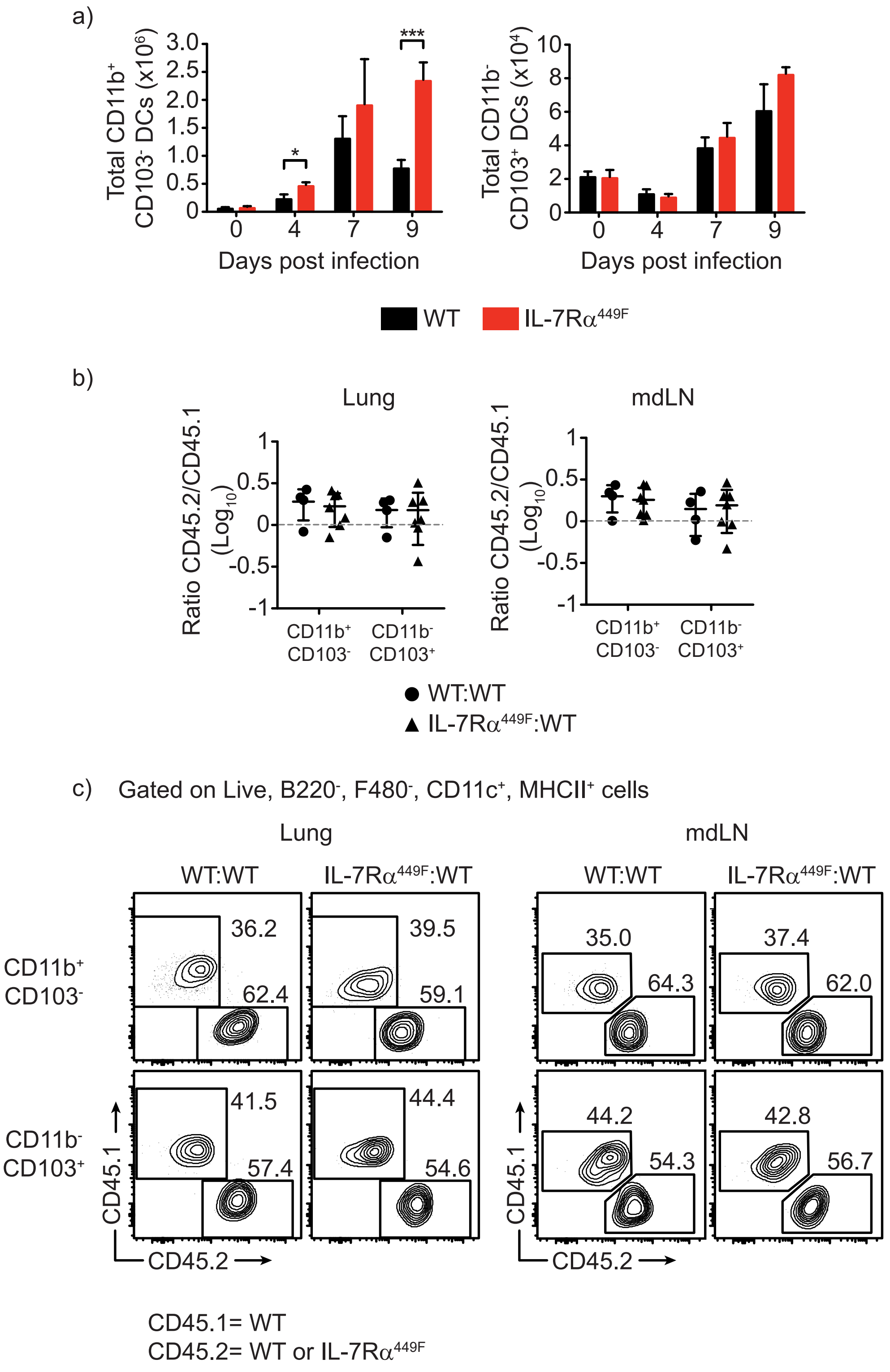

Supplementary Figure 2. Loss of IL-7Rα signaling leads to increased accumulation of $CD11b^+$ $CD103^-$ dendritic cells in the lungs. (a) Flow cytometric analysis showing total number of $CD11b^+$ $CD103^-$ (left) and $CD11b^-$ $CD103^+$ (right) dendritic cells in the lungs of WT and IL-7Rα^{449F} mice at indicated days post infection presented as a bar graph. Gated within Live $CD45^+$, $B220^-$, $F4/80^-$, $CD11c^{hi}$, $MHCII^{hi}$, $CD11b^{+/-}$ and $CD103^{-/+}$. (b, c) Bone marrow chimera analysis of lung $CD11b^+$ $CD103^-$ and $CD11b^-$ $CD103^+$ dendritic cells presented as (b) bar graphs and (c) FACS plots. (b) Data presented as ratio of the CD45.2:CD45.1 and plotted with log10 transformation to normalize skewed data points. Data are representative of two experiments with n=4-6 per genotype. ***P<0.001 as determined by two-tailed Student's t-test.

Supplementary Figure 3

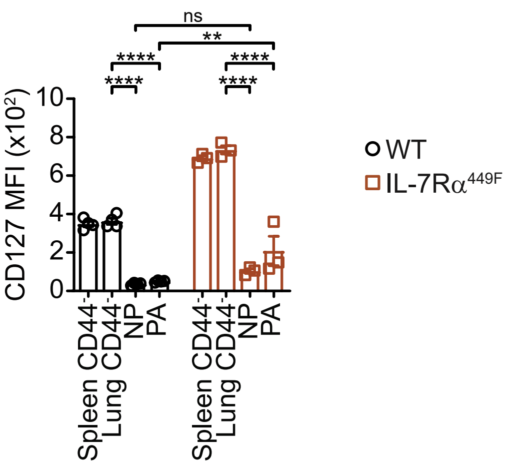

Supplementary Figure 3. CD127 expression by median fluorescence intensity (MFI) in spleen $CD44^-$, lung $CD44^-$ and lung influenza-specific CD8 T cells of WT and IL-7Rα^{449F} mice. Data are representative of two experiments with n=3-5 per genotype. **P<0.01 and ****P<0.0001 as determined by one-way ANOVA.
